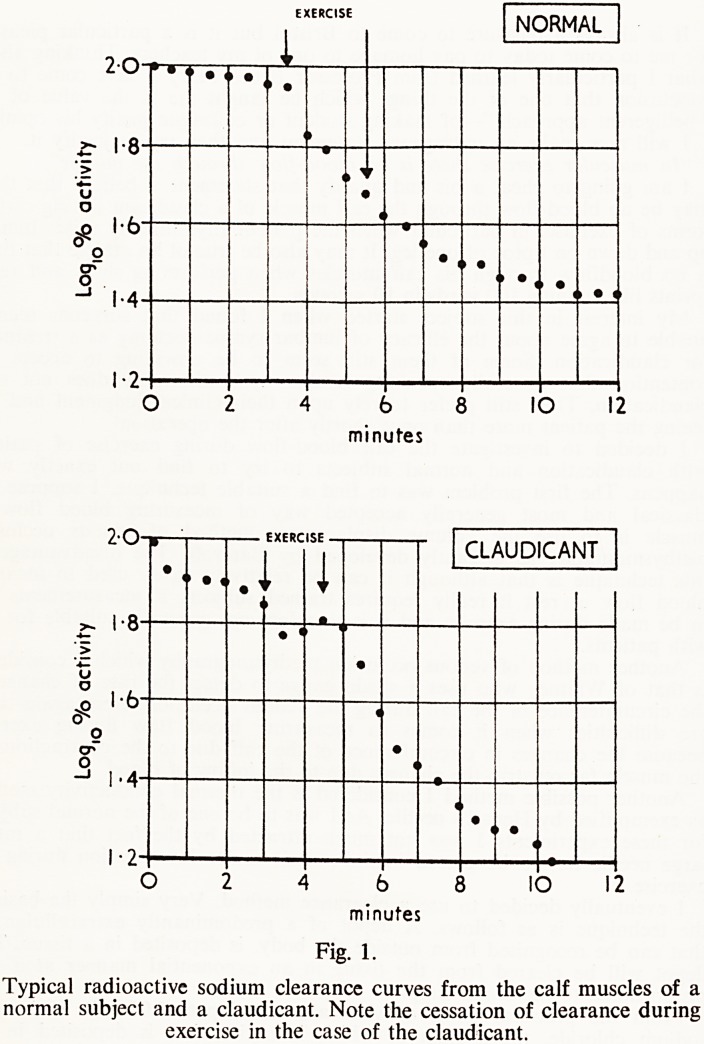# Claudication

**Published:** 1969-10

**Authors:** D. N. Walder


### CLAUDICATION

D. N. Walder

It is always a pleasure to come to Bristol but it is a particular pleasure for me to come today to pay homage to one of my teachers. Thinking about what I particularly learned from Professor Bruce Perry I have come to the conclusion that one of the things which he taught me is the value of the " belligerent approach "—of making student or colleague justify his opinion.

I will now make an outrageous statement and then try to justify it.

*"In muscular exercise there is no blood-flow through the muscle".*

I am going to cheat a bit and qualify that statement. I believe that there may be no blood-flow through the calf muscle of a claudicant during certain forms of exercise, in particular when the patient rhythmically raises himself up and down on tiptoe of one leg. It may also be true of an athlete that there is no bloodflow through his calf muscles when performing short and rapid sprints like running 100 yards in 10 seconds.

My interest in this subject started when I found that surgeons seemed unable to agree about the efficacy of lumbar sympathectomy as a treatment for claudication. Some of them still seem to be unwilling to accept my contention that generally speaking lumbar sympathectomy does not cure claudication. They still prefer to rely upon their clinical judgment and not seeing the patient more than once shortly after the operation!

I decided to investigate the calf blood-flow during exercise of patients with claudication and normal subjects to try to find out exactly what happens. The first problem was to find a suitable technique. I suppose the classical and most generally accepted way of measuring blood flow in muscle is to use the volume displacement method of venous occlusion plethysmography so elegantly developed by Barcroft. The disadvantage of this technique is that although it can be relatively easily used to measure blood flow at rest it really requires trained subjects if measurements are to be made during exercise and it is therefore not generally suitable for use with patients.

Another method of venous occlusion plethysmography which I considered is that of Whitney who uses a strain gauge to detect the rate of change in the circumference of the calf during the venous occlusion. Here again there are difficulties when it comes to measuring blood flow during exercise because the changes in circumference of the calf due to the contractions of the muscle far outstrip the changes due to the inflow of blood.

Another possible method I considered is the thermal conductivity method as exemplified by Hensel's needle. As I was to be one of the normal subjects for these experiments I was not much attracted by the fact that a rather large needle has to be placed in the muscle under investigation during the exercise.

I eventually decided to use a clearance method. Very simply the basis of the technique is as follows. A depot of a predominantly extracellular ion that can be recognised from outside the body, is deposited in a tissue. This depot will be cleared from the tissue in an exponential manner at a rate proportional to the blood supply.

From the practical point of view a small depot (0.05 ml) of isotonic sodium chloride, the sodium of which is radioactive, is deposited in the muscle to be tested, in this case the calf. The radioactivity of the depot is detected by a geiger counter tube applied to the calf. If the rate of clearance of the sodium is compared with the changes in blood flow in the calf as

Fig. 1.

Typical radioactive sodium clearance curves from the calf muscles of a normal subject and a claudicant. Note the cessation of clearance during exercise in the case of the claudicant.

determined by Barcroft's plethysmographic technique, it can be seen that the two methods give similar results. So now we have established that the radioactive sodium clearance method is at least qualitatively justifiable.

Let us now look at the blood flow before, during and after exercise in a patient known to claudicate. A surprising fact comes to light. Sodium clearance ceases during exercise and therefore there is presumably no blood flow occurring during this time (Fig. 1).

Can this really be so? What is the explanation? Calf muscles are spindle shaped, and this is important because we do not claudicate in strap muscles where the muscle fibres are parallel to the pull of the muscle. The muscle fibres of a calf muscle, however, lie in a different direction from the direction of pull of the Achilles tendon. If the direction of pull of the muscle fibres is considered to be the vector, one of the resultant forces will be the pull on the Achilles tendon and there will have to be a second resultant force in a horizontal direction. This second resultant force will give rise to an internal pressure in the muscle during contraction.

It therefore becomes evident that if the pressure in the nutrient vessel, which in the case of the calf comes from the popliteal artery, is less than the pressure developed in the muscle during exercise then no blood can enter the muscle during contraction.

It has been shown that the pressure in the popliteal artery in patients with peripheral vascular disease when measured at operation may be markedly below normal and instead of being 120/80 mm Hg it may be 40/20 mm Hg or even lower.

It has been possible by means of a tocodynamometer to estimate the pressure developed in calf muscle during contraction. It can be shown that just standing on one leg results in the development of a pressure in the calf muscle of the order of 40 mm Hg and that during running pressures of more than 150 mm Hg can be developed.

We have been able to show that in a claudicant, just standing on tiptoe on one leg will bring about a cessation of blood flow in his calf muscle.

These observations have led me to propose the following hypothesis: that claudication occurs when the pressure developed in the calf muscle during contraction is greater than the pressure of the blood in the nutrient vessel to the muscle.

If this hypothesis is correct then it should be possible to convert a normal person into a claudicant by increasing the pressure developed in the calf muscle during exercise until it becomes greater than the blood pressure existing in the nutrient vessel to that muscle. In this case no calf muscle blood-flow would occur during exercise and the person would claudicate. Conversely it should be possible to convert a claudicant into a normal person by decreasing the pressure developed in the calf muscle during exercise until it becomes less than the blood pressure existing in the nutrient vessel to that muscle. In this case calf blood-flow would occur during exercise and the patient would not claudicate.

By arranging that the subject lifts known weights instead of his body when rising up and down on his toes it has been possible to increase the weight lifted until a point is reached when the pressure developed in the calf muscle of a normal subject is such that blood flow ceases during the exercise and conversely it has been possible to decrease the weight until a point is reached when the pressure developed in the calf muscle of a claudicant is such that blood flow can be maintained during the exercise.

The radioactive sodium clearance technique has been developed into an objective test to determine whether or not a patient claudicates. If he is able to maintain a clearance of sodium from the calf muscle during a period of exercise in which he lifts his body weight on one leg up and down in time to a metronome set at 60 beats per minute then he is said to have an adequate blood supply to the calf muscles; but if he is not able to do this and there is a cessation of clearance of sodium during the exercise then the patient is said to be a claudicant.

Such an objective test is of considerable clinical value. For instance, a patient who has been claudicating can be tested before and after the removal of an arterial obstruction when it will be found that the pre-operative typically abnormal picture of no blood-flow during exercise is converted into the typically normal picture with considerable blood-flow during the exercise. In passing it should be said that I know of no instance in which an abnormal radioactive sodium clearance from a calf muscle has been converted to a normal pattern following lumbar sympathectomy.

An interesting application of this test is that since the medial and lateral heads of the gastrocnemius muscle are independently supplied by separate nutrient arteries it is possible to show that in some patients one head of the gastrocnemius muscle may be suffering from vascular insufficiency during exercise whilst the other head is quite normal. In such circumstances it is sometimes justifiable to treat the patient by the simple process of denervating the head giving rise to the pain of claudication.

It may also be a useful adjunct to radiological examination in that occasionally an arteriogram appears to show a normal arterial tree when in fact there is an obstruction sufficient to result in a drop in the blood pressure of the nutrient vessel to the calf muscles with a consequent cessation of blood flow and onset of claudication during exercise.

Pressure may be developed in the calf muscle not only as a result of the forces developed when the muscle fibres contract but also if the muscle becomes too big for the fascial sheath in which it lies. Young men who suddenly take up intensive athletic training sometimes develop an excessive hypertrophy of the gastrocnemius muscle so that when the muscle changes shape and position during contraction it is squeezed by the tight sheath and its internal pressure becomes higher than that in the nutrient vessel supplying it, and claudication results.

One rare cause of intermittent claudication is the presence of an abnormally placed popliteal artery which instead of lying between the two heads of the gastrocnemius muscle passes through one head. Every time the muscle contracts it squeezes the popliteal artery and blocks its own blood supply. This condition is difficult to spot but can be demonstrated quite nicely using the radioactive sodium clearance technique.

One question which is always asked when I claim that during contraction the pressure developed in the calf muscle may be so high that no blood can get into the muscle is, what happens during relaxation? Surely the pressure in the muscle must then drop to a sufficiently low level to allow blood to flow in and this should be sufficient to keep the muscle oxygenated during the exercise.

Whether or not this is a valid argument depends on the volume of blood per unit volume of muscle which is able to flow in during the relaxation phase and this will be related to the duration of the phase and to the rate of flow of blood.

Using a portable apparatus it has been possible to show that whereas I can maintain a blood flow through my calf muscles when running slowly I am not able to maintain a blood-flow through my calf muscles when running fast. As soon as I speed up, the relaxation phase is too short for a sufficient quantity of blood to get into the muscle. Muscle is like a sponge. When it contracts all the blood is squeezed out. If the volume of blood that can flow into the muscle during the relaxation phase between contractions is insufficient to fill the arterial tree then no blood will get to the capillary bed because the next contraction will force the blood back.

To summarize then, I would like to suggest that the radioactive sodium clearance technique is a useful objective test to determine whether or not a patient is able to maintain a blood supply to his muscle during exercise. If he can then he will not claudicate; if he cannot then he will claudicate.

### References

Barcroft, H. and Dornhorst, A. C. (1949) J. Physiol. *109*, 402.

Hensel, H., Ruef, J. and Golenhofen, K. (1954) Pflugers Arch. ges Physiol. *259*, 267.

Molyneux, L., Turnbull, J. and Walder, D. N. (1958) J. Sci. Inst. *35*, 259.

Smyth, C. N. (1957) J. Obstet. Gynaec. *64*, 59.

Walder, D. N. (1953) Clin. Sci. *12*, 153.

Walder, D. N. (1954) Radioisotope Conference, *1*, 174.

Walder, D. N. (1955) Clin. Sci., *14*, 303.

Walder, D. N. (1956) Med. Illustrated, *10*, 229.

Walder, D. N. (1958) Brit. Med. J. *i*, 255.

Walder, D. N. (1961) J. Physiol. *159*, 70P.

Walder, D. N. (1964) Vth International Congress of Angiology, Pergamon Press, Oxford. p.3.

Walder, D. N. (1968) 'Circulation in Muscle' Ed. O. Hudlicka, Pergamon Press, London. p. 173.

Walder, D. N. (1968) 'Blood Flow through Organs and Tissues' Ed. Bain and Harper. Livingstone, London. p. 383.

Whitney, R. J. (1953) J. Physiol. *121*, 1.

## Figures and Tables

**Fig. 1. f1:**